# Supplementary material for: Polarity signaling ensures epidermal homeostasis by coupling cellular mechanics and genomic integrity
Source: Nat Commun. 2019 Jul 29;10:3362. doi: 10.1038/s41467-019-11325-3 (PMC6662827; doi:10.1038/s41467-019-11325-3)
Supplement: Supplementary file 1 — Supplementary Information [file 41467_2019_11325_MOESM1_ESM.pdf]

Polarity signaling ensures epidermal homeostasis by coupling cellular mechanics and genomic integrity

Dias Gomes, Letzian et al.

Supplementary Information

- Supplementary Figure 1
- Supplementary Figure 2
- Supplementary Figure 3
- Supplementary Figure 4
- Supplementary Figure 5
- Supplementary Figure 6
- Supplementary Figure 7
- Supplementary Figure 8
- Supplementary Figure 9
- Supplementary Figure 10
- Supplementary Figure 11
- Supplementary Figure 12
- Supplementary Table 1
- Supplementary Table 2
- Supplementary Table 3

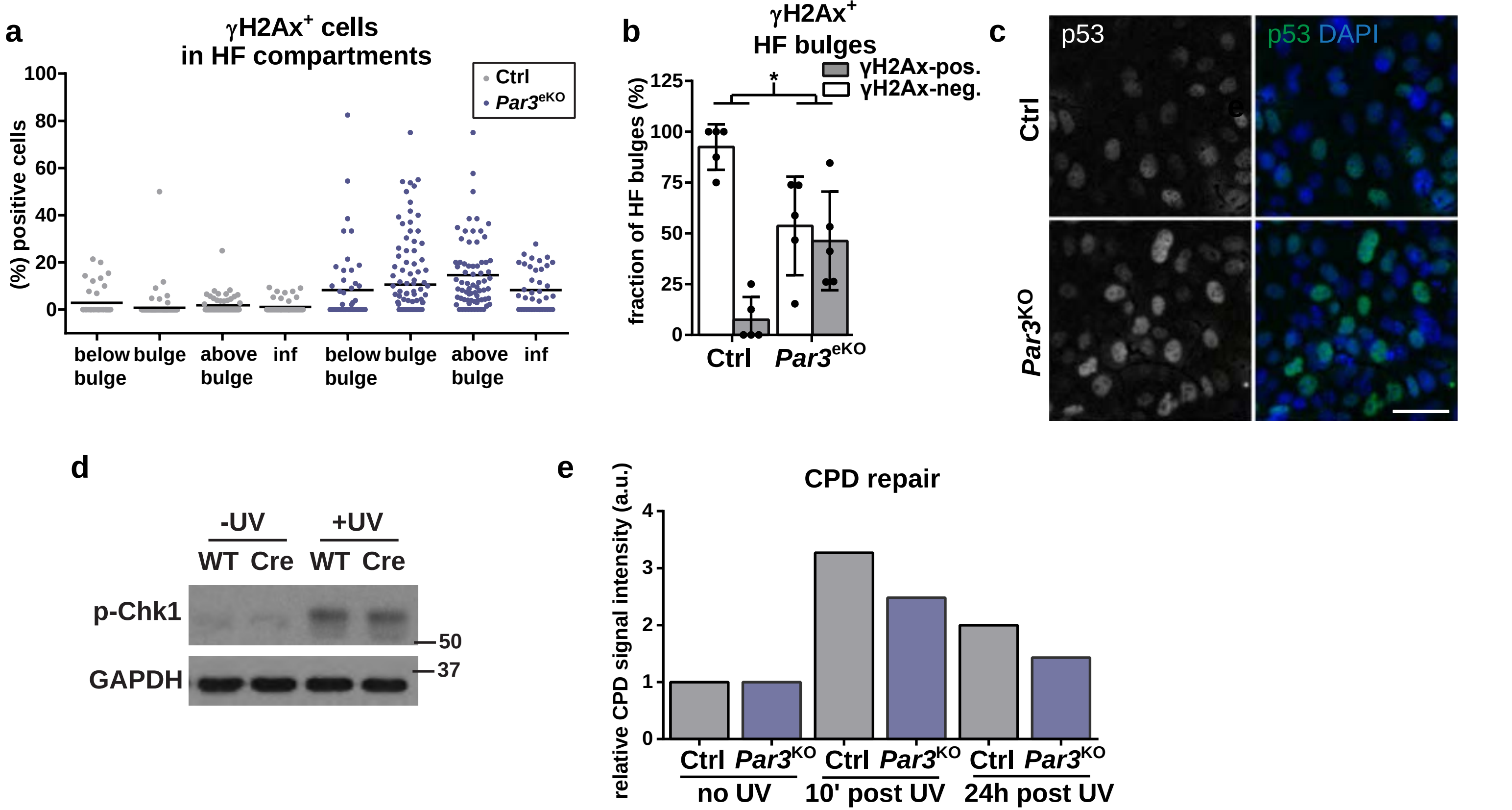

Supplementary Figure 1. Increased DNA damage upon Par3 loss. **(a)** Percentage of γH2Ax-positive cells per HF compartment from *Par3A* knockout (*Par3*^{eKO}: K14Cre/+;*Par3*fl/fl) and control mice (K14Cre/+). Graph shows percentage of γH2Ax-positive cells in individual compartments pooled from 5 animals per genotype. Compartments are detailed in Figure 1. Bar represents mean. **(b)** Percentage of HF bulges (K15^{high}) with cells positive for γH2Ax. n=5 mice, *: p=0.0298**;** mean±SD, two-way ANOVA/Sidak's multiple comparisons test. **(c)** p53 (green) immunofluorescence micrographs of primary *Par3*^{KO} and control keratinocytes. DAPI (blue) was used to counterstain nuclei. Scale bar: 50µm. Micrographs were subjected to rolling ball background subtraction, radius=50µm, and are representative for three independent experiments **(d)** Immunoblot analysis of pChk1 in control (K14Cre/+) and wildtype keratinocytes, either non-treated or UV-B treated (100mJ/cm^2). **(e)** DNA repair assay. Cyclobutane pyrimidine dimer (CPD) signal intensity from slot blot experiment normalized to total DNA. DNA was isolated from *Par3*^{KO} and control keratinocytes lysed at indicated time-points after UV-B irradiation (3mJ/cm^2). Data are representative for 3 individual performed experiments. Western blot micrographs have been cropped. Abbreviations used: Ctrl, control; inf, infundibulum; HF, hair follicle; WT, wild-type.

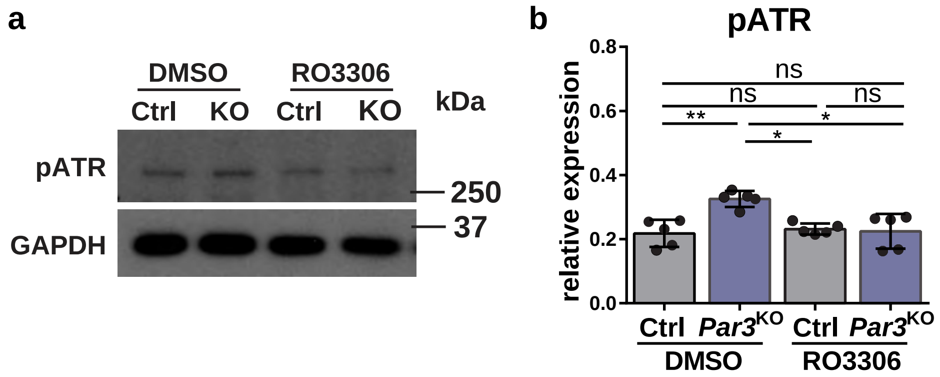

Supplementary Figure 2. Mitosis-dependent increase of DNA damage signaling upon Par3 loss. **(a)** Immunoblot analysis of pATR in primary Par3-deficient and control keratinocytes after treatment with Cdk1 inhibitor RO3306 (15µM). GAPDH served as loading control. **(b)** Quantification of (a). pATR levels were first normalized to GAPDH and then expressed as relative values. n=5 biologically independent samples; mean±SD; two-way ANOVA/Tukey's multiple comparisons test *: p=0.0227 (*Par3*^{KO}/DMSO vs Ctrl/RO3306); *: p=0.0145 (*Par3*^{KO}/DMSO vs *Par3*^{KO}/RO3306); **: p=0.0095 (Ctrl/DMSO vs *Par3*^{KO}/DMSO). Cropped immunoblot data are shown. Abbreviations used: Ctrl, control; ns, non-significant; KO, *Par3*^{KO}.

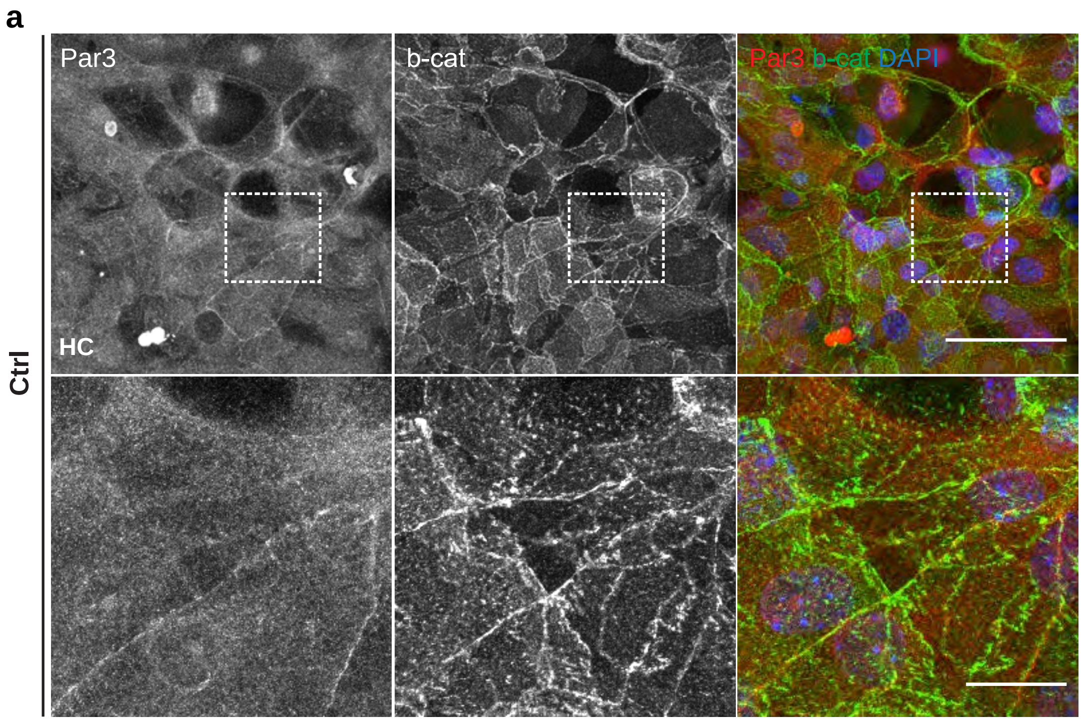

Supplementary Figure 3. Par3 localizes to keratinocyte cell-cell contacts. **(a)** Immunofluorescence micrographs of control keratinocytes stained for Par3 (red) and beta-catenin (green). DAPI is shown in blue, individual channels are displayed in gray. Scale bar: 80µm. Lower panel shows magnification (dashed box) of junctional staining for the indicated channels. Scale bar: 40µm. Micrographs are representative for 3 individual experiments. Image intensity was enhanced for better visualization. Abbreviations used: Ctrl, control; HC. High calcium; b-cat, beta-catenin.

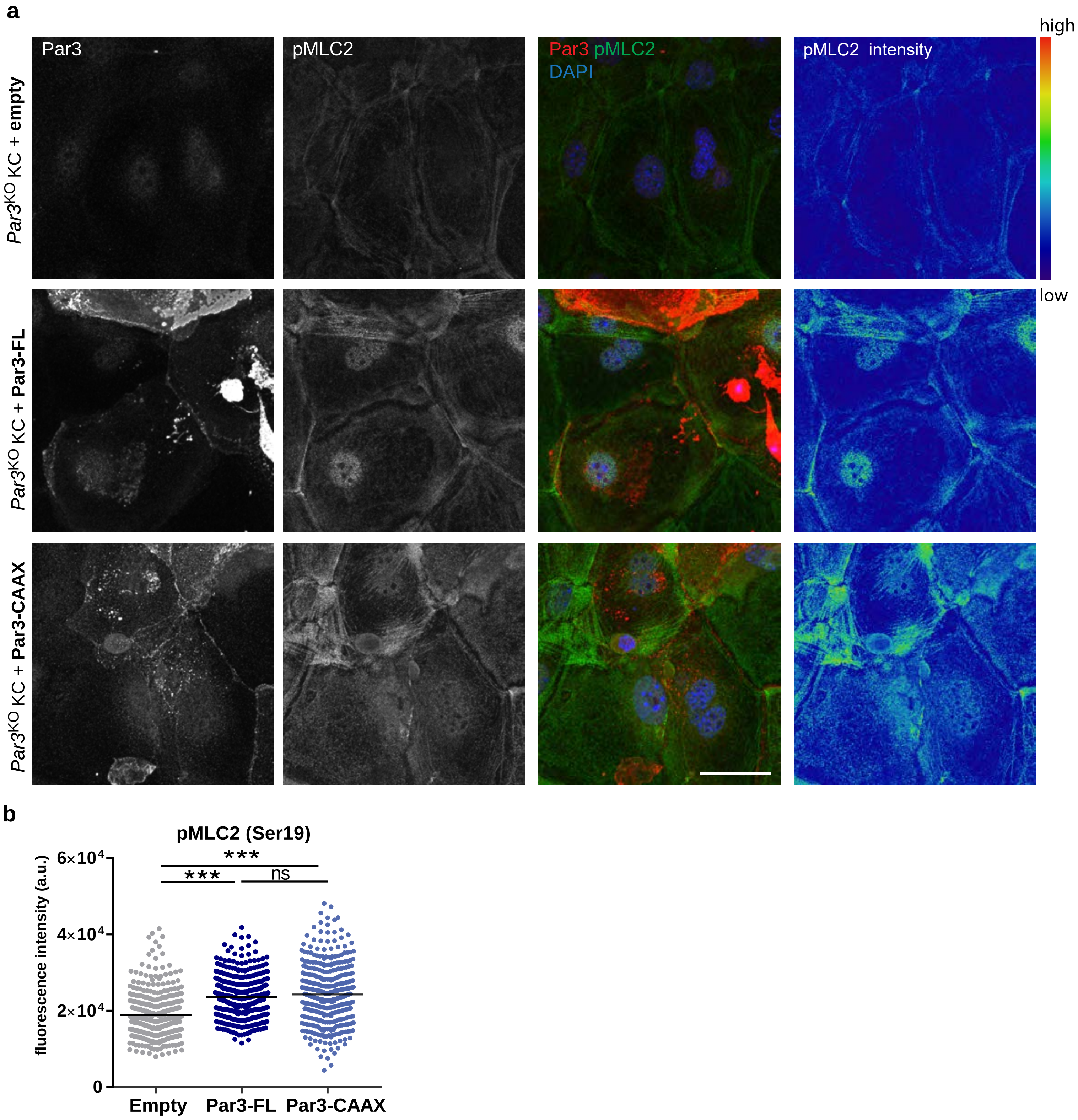

Supplementary Figure 4. Ectopic Par3 expression restores junctional pMLC2. **(a)** Immunofluorescence micrographs of *Par3*^{KO} keratinocytes transiently transfected with empty vector, Par3 full-length (Par3-FL), or membrane-targeted Par3-CAAX, and stained for Par3 (red) and pMLC2 (green). Panels show individual channels (gray), merge, full-range physics look-up table and pseudo-color scale. Scale bar: 40μm. **(b)** Quantification of pMLC2 immunoreactivity at cell-cell junctions, intensity in arbitrary units; n=433 cells pooled from three independent experiments; ***: $p<0.0001$, two-sided Mann–Whitney U test, bar represents mean. Abbreviations used: ns, non-significant; KC, keratinocytes.

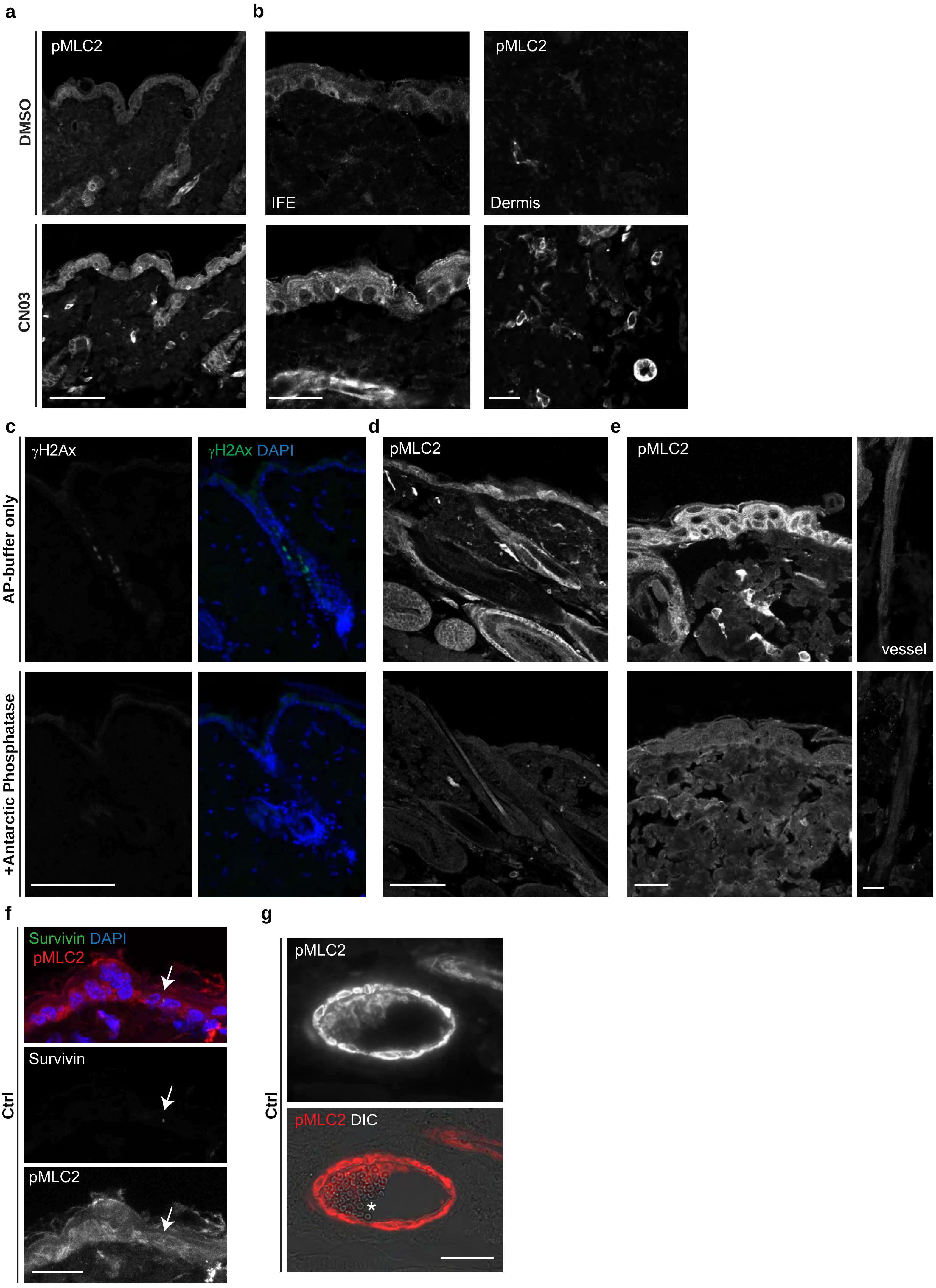

a
b
pMLC2
DMSO
IFE
Dermis
CN03
c
d
e
γH2Ax
γH2Ax DAPI
AP-buffer only
+Antarctic Phosphatase
vessel
f
g
Survivin DAPI
pMLC2
Survivin
Ctrl
pMLC2 DIC
*

Supplementary Figure 5. Validation of pMLC2 antibody in immunohistochemistry of adult skin. **(a-b)** Immunohistochemistry for pMLC2 (Ser19) (gray) (Cell Signaling Technologies #3675). Skin explants of control murine back-skin were treated with DMSO (top panel) or 5µg/ml CN03 (bottom panel). **(a)** Overview micrographs (20x objective). Scale bar: 40µm. **(b)** Higher magnification (63x objective) of IFE (left panel) and dermis (right panel) showing fibroblast-derived signals. Scale bars: 20µm (IFE), 20µm (dermis). Micrographs are representative for two biological replicates. **(c-e)** Dephosphorylation of epitopes in skin sections. Back-skin cross sections were treated with Antarctic phosphatase (AP) in AP-buffer, or with AP-buffer only as control. **(c)** Representative micrographs of γH2Ax (Ser139) (green) (Cell Signaling Technologies #9718) signals in control sections (upper panel), and following AP treatment (lower panel). 20x objective, scale bar: 40µm. **(d,e)** Representative micrographs of pMLC (Ser19) (gray) (Cell Signaling Technologies #3675) immunostaining in sections incubated with AP buffer only (upper panel), or with phosphatase (lower panel). **(d)** Overview micrographs (20x objective, scale bar: 40µm). **(e)** Higher magnification micrographs of skin epithelium (left panel) and blood vessels (right panel) incubated without or with AP. 63x objective, scale bar: 20µm (skin epithelium), 10µm (blood vessel). Micrographs are representative for four independent experiments. **(f)** Co-immunostaining of mouse α-pMLC2 (Ser19) (red) (Cell Signaling Technologies #3675) and rabbit α-Survivin (green) (Cell Signaling Technologies #2808) in adult murine skin cross-section. DAPI is shown in blue, individual channels are shown in gray. Scale bar: 20µm. Micrographs are representative for five biological replicates. **(g)** Immunohistochemistry of adult murine skin cross-sections stained for pMLC2 (Ser19) (top panel gray, bottom panel red) (Cell Signaling Technologies #3675). Micrographs show pMLC immunostaining in smooth muscle layers of large skin blood vessels individually (upper panel) and merged with DIC (lower panel). Asterisk (*) marks erythrocytes within the blood vessel. 63x objective, scale bar: 50µm. Micrographs are representative for three biological replicates. Abbreviation used: Ctrl, control; IFE, interfollicular epidermis; HF, hair follicle; DIC, differential interference contrast.

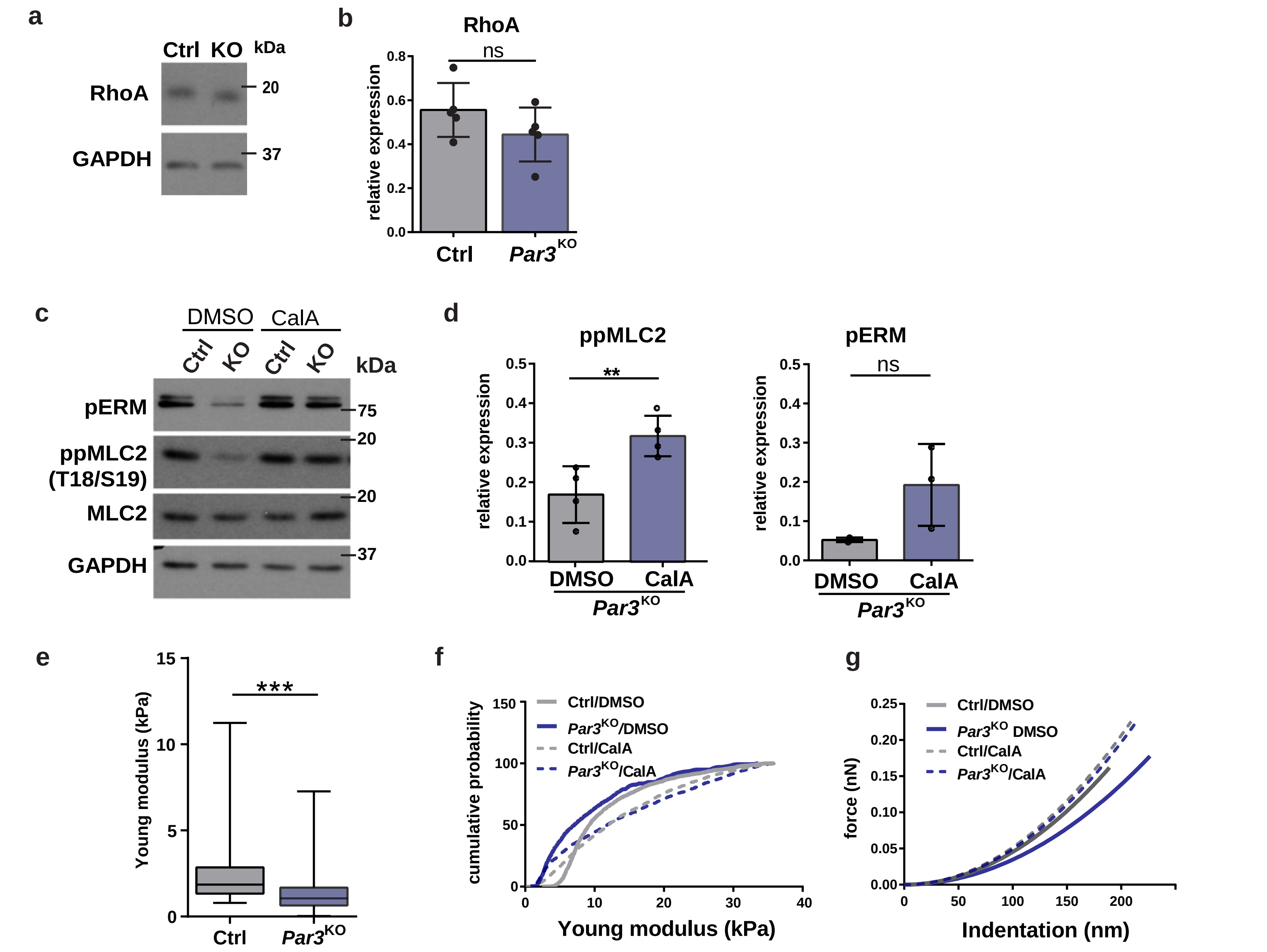

Supplementary Figure 6. Par3 loss impairs myosin activity and viscoelastic properties of keratinocytes. **(a)** Immunoblot analysis of RhoA in keratinocyte lysates. GAPDH served as loading control. **(b)** Quantification of (a). RhoA levels were first normalized to GAPDH and then expressed as relative values. n=5 biologically independent samples; mean±SD; paired two-tailed Student's t-test, ns: p=0.3661. **(c)** Immunoblot analysis of pERM, ppMLC2 (Thr18/Ser19), and MLC2 in keratinocyte lysates following 24h CalA (1nM) treatment. GAPDH served as loading control. Micrograph is representative for 3 individual experiments. **(d)** Quantification of (c). ppMLC2 (Thr18/Ser19) levels were normalized to GAPDH and then expressed as relative values. n=4 biologically independent samples; mean±SD; paired two-tailed Student's t-test, **: p=0.0081. pERM levels were first normalized to GAPDH and then expressed as relative values. n=3 biologically independent samples, paired two-tailed Student's t-test, ns: p=0.1412 mean±SD. **(e)** Young Modulus box-plot based on force indentation spectroscopy with a spherical tip (Ctrl: n=467 measurements, *Par3*^{KO}=594 measurements, pooled from three independent experiments ***, p<0.0001, Mann-Whitney U-test, box plots show minimum (boundary of lower whisker), 25th percentile (lower boundary of box), median (center line), 75th percentile (upper boundary of box) and maximum (boundary of upper whisker). **(f)** Cumulative probability of Young Moduli extracted from Hertzian fits from force spectroscopy indentations (n=2000 force curves, pooled from three independent experiments). **(g)** Indentation force plots showing mean Hertzian fits (n=8 representative force indents per condition). Cropped immunoblot data are shown. Abbreviations used: Ctrl, control; KO, *Par3*^{KO}; ns, non-significant; CalA, Calyculin A.

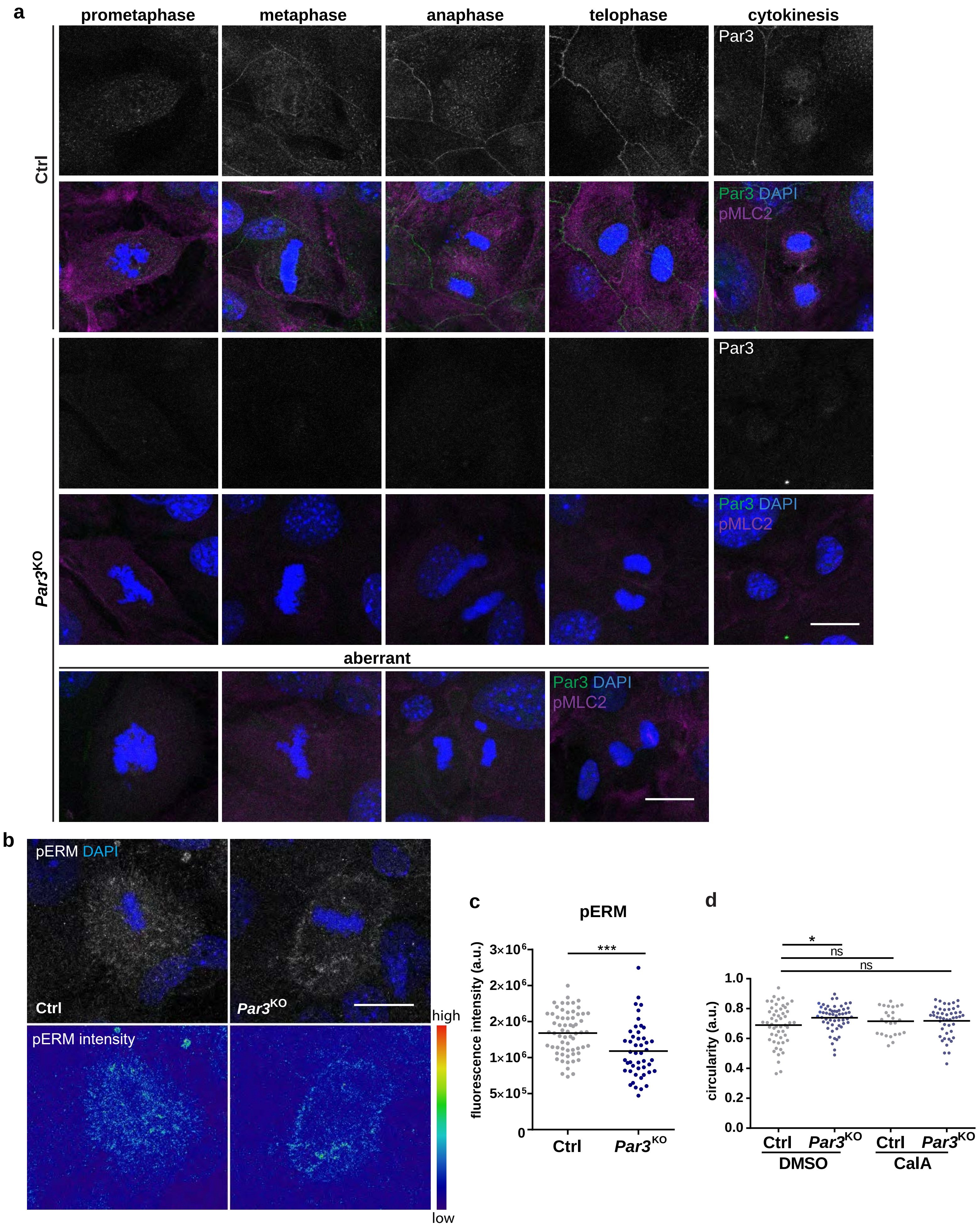

a
prometaphase
metaphase
anaphase
telophase
cytokinesis
Ctrl
Par3
Par3 DAPI
pMLC2
Par3KO
aberrant
b
pERM DAPI
Ctrl
Par3KO
pERM intensity
high
low
c
pERM
fluorescence intensity (a.u.)
3×10^6
2×10^6
1×10^6
5×10^5
0
***
Ctrl
Par3KO
d
circularity (a.u.)
1.0
0.8
0.6
0.4
0.2
0.0
*
ns
ns
Ctrl
Par3KO
Ctrl
Par3KO
DMSO
CalA

Supplementary Figure 7. Localization of Par3, phosphorylated MLC2 and phosphorylated ERM during mitosis. **(a)** Immunofluorescence analysis of Par3 (green) and pMLC2 (magenta) in primary *Par3*^{KO} and control keratinocytes during mitosis. DAPI is shown in blue, individual channels are displayed in gray. Micrographs show Par3 and pMLC2 localization during different phases of mitosis and are representative for three individual experiments. Scale bar: 40µm **(b)** Immunofluorescence micrographs of pERM (gray) in primary murine keratinocytes, physics look-up table and pseudo-color scale.Scale bar: 40µm. **(c)** Quantification of pERM immunoreactivity, intensity in arbitrary units; n=64 cells (Ctrl), n=47 cells (*Par3*^{KO}) cells pooled from three independent experiments; ***: p<0.0001, two-sided Mann–Whitney U-test, bar represents mean. **(d)** Mitotic spindle circularity measurements in Ctrl and *Par3*^{KO} keratinocytes after DMSO or CalA (1nM) treatment (Ctrl/DMSO: n=53 cells, *Par3*^{KO}/DMSO: n=55 cells, Ctrl/CalA: n=25 cells, *Par3*^{KO}/CalA: n=47 cells, pooled from four independent experiments, *: p=0.0432 (Ctrl/DMSO vs. *Par3*^{KO}/DMSO), two-tailed one-way ANOVA, bar represents mean. Abbreviations used: Ctrl, control; ns, non-significant; CalA, Calyculin A.

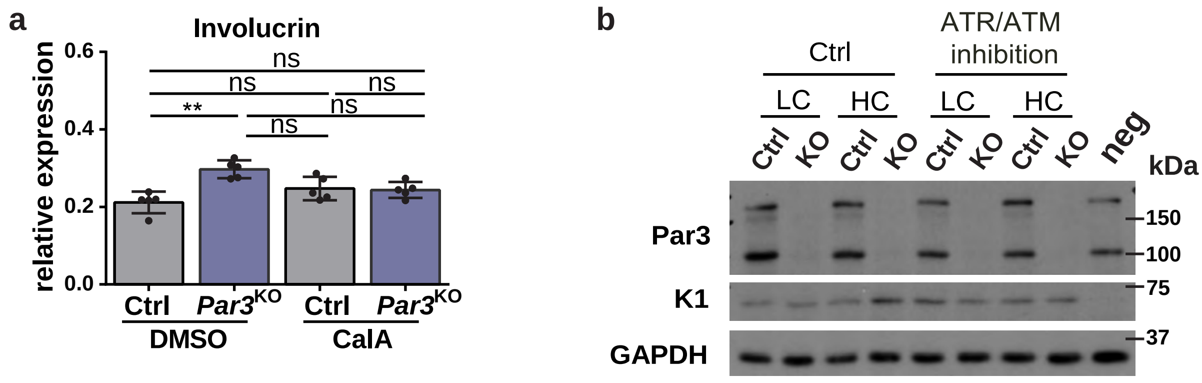

Supplementary Figure 8. CalyculinA or ATM/ATR inhibition normalize epidermal differentiation in the absence of Par3. **(a)** Quantification of immunoblot analysis for Involucrin expression in keratinocyte lysates following 48h CalA treatment. GAPDH served as loading control. Involucrin levels were first normalized to GAPDH and then expressed as relative values. n=5 biologically independent samples; mean±SD; **: p=0.0031 (Ctrl vs. *Par3*^{KO}, DMSO-treated); mean±SD; two-way ANOVA/Tukey's multiple comparisons test. **(b)** Immunoblot analysis for Keratin1 upon ATR/ ATM inhibition. GAPDH served as loading control. Cropped immunoblot data are shown. Abbreviations used: Ctrl, control; KO, *Par3*^{KO}; ns, non-significant; CalA, Calyculin A; neg, negative control; ATM, Ataxia telangiectasia mutated; ATR, ATM and RAD3-related.

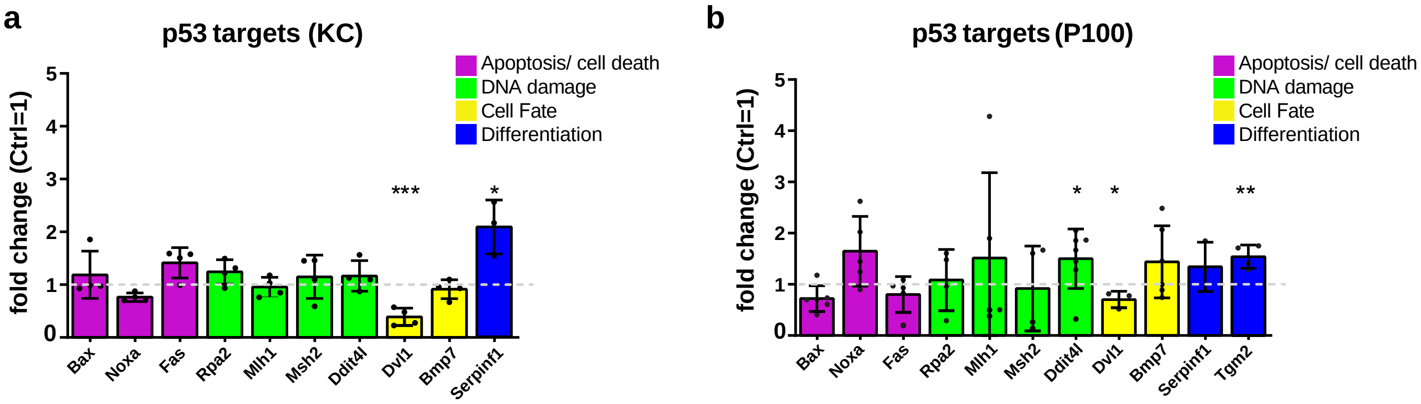

Supplementary Figure 9. Expression of selected p53 targets in Par3-deficient keratinocytes and epidermis. **(a, b)** qRT-PCR analysis of p53 target genes in primary *Par3*^{KO} and control keratinocytes (a) and P100 skin epidermis of *Par3*^{eKO} and control mice (b). Target genes are categorized in apoptosis and cell death (magenta), DNA damage (green), cell fate (yellow) and differentiation (blue) as indicated. Gene expression was normalized to HPRT (loading control) and then normalized to control keratinocytes or epidermis, respectively. n=3(Serpinf1, in a; Dvl1, Serpinf1, in b) n=4(Bax, Noxa, Fas, Rpa2, Mlh1, Msh2, Ddit4l, Dvl1, Bmp7, in a; Rpa2, Msh2, Tgm2, in b), n=5 (Noxa, Fas, Mlh1, in b), n=6 (Bax, Bmp7, in b), n=7 (Ddit4l, in b), ***: p=0.0003(Dvl1, in a), *: p=0.0216(Serpinf1, in a) *: p=0.0419(Ddit4l, in b), *: p=0.0327(Dvl1, in b), **: p=0.0031(Tgm2, in b), (a): n= biologically independent samples, (b) n= mice, mean±SD; unpaired two–tailed Student's t-test. Abbreviations used: Ctrl, control; KC, keratinocytes.

Figure 1h

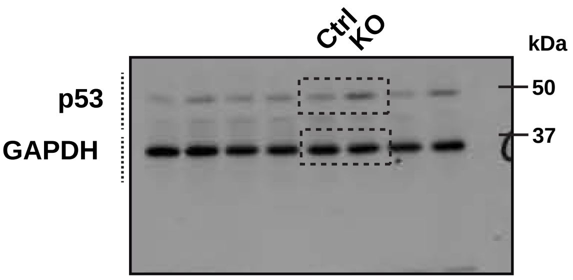

Figure 1j

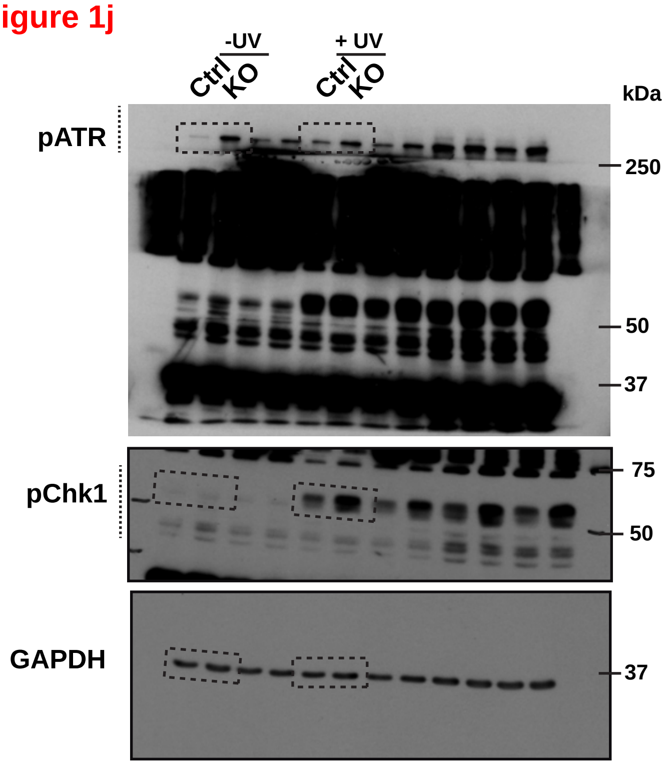

Figure 2f

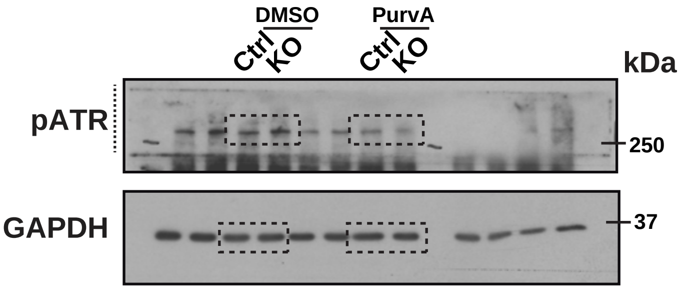

Figure 3h

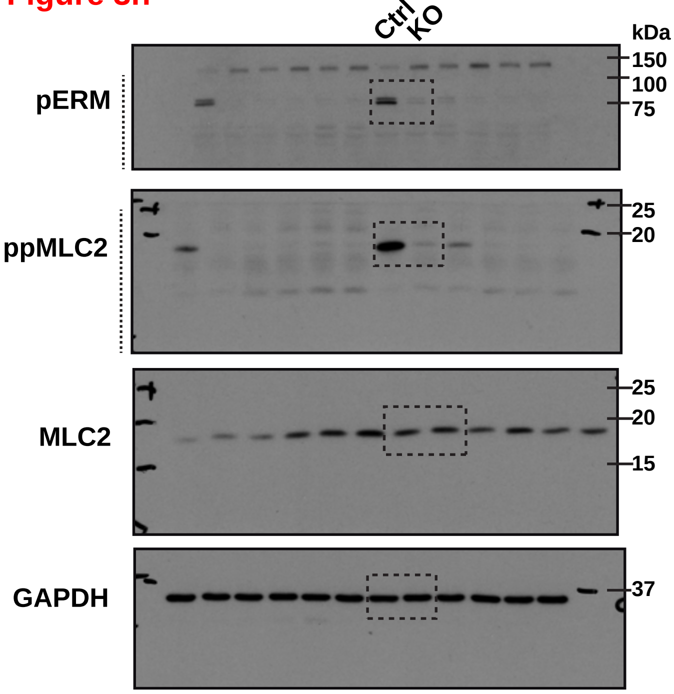

Figure 3k

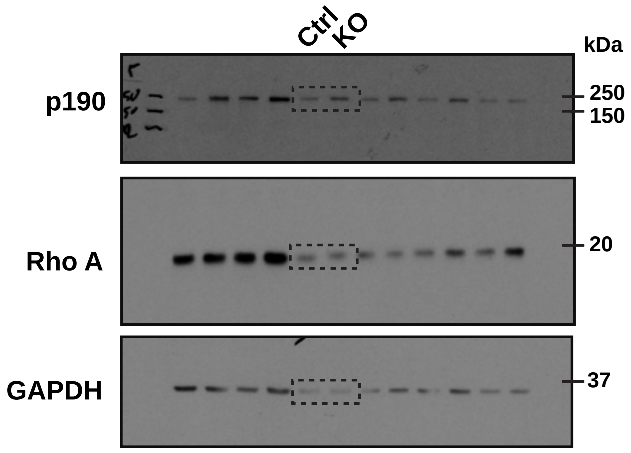

Figure 6e

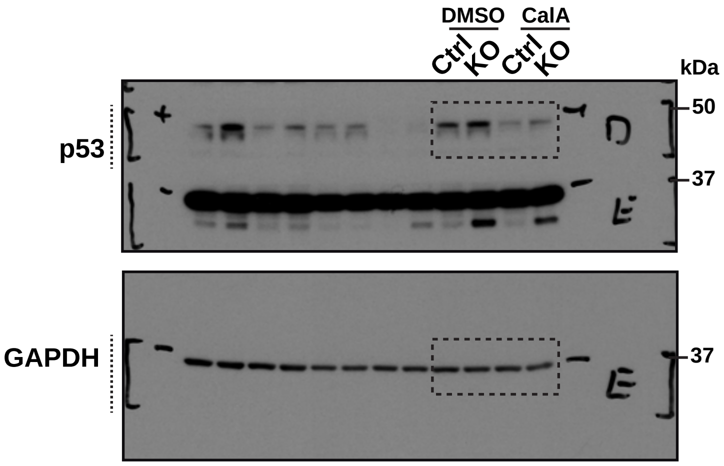

Figure 6h

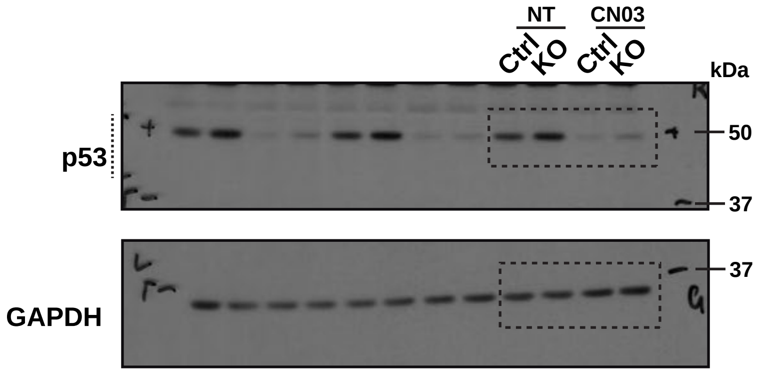

Supplementary Figure 10. Original western blots of the manuscript (1 of 3). Left vertical lines refer to the part of the membrane that was incubated with the antibody indicated next to it.

Figure 7a

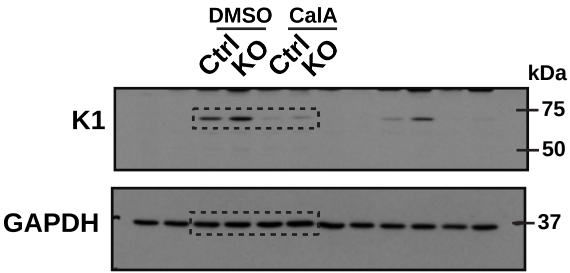

Figure 7c

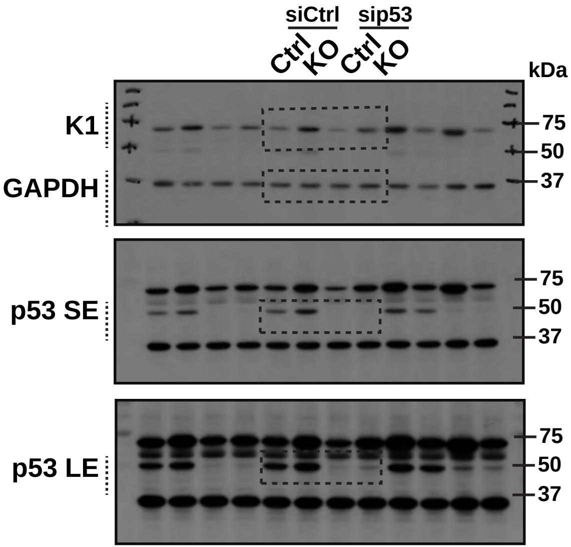

Supplementary Figure 11. Original western blots of the manuscript (2 of 3). Left vertical lines refer to the part of the membrane that was incubated with the antibody indicated next to it.

Supplementary Figure 1d

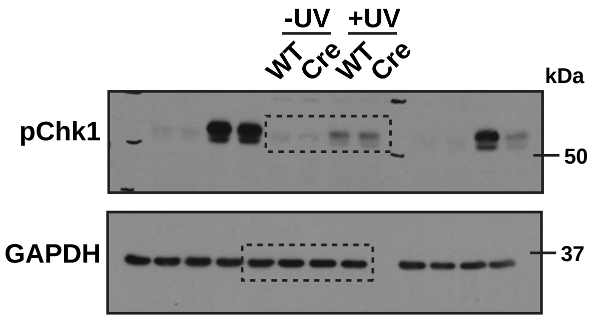

Supplementary Figure 2a

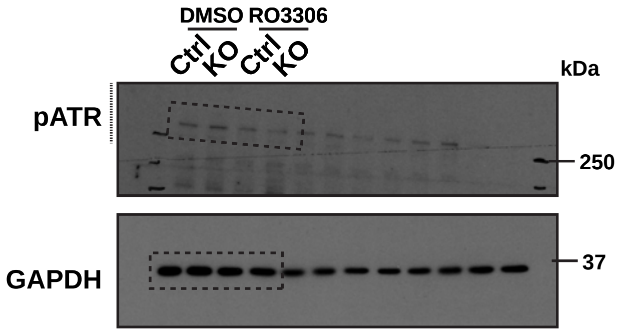

Supplementary Figure 6a

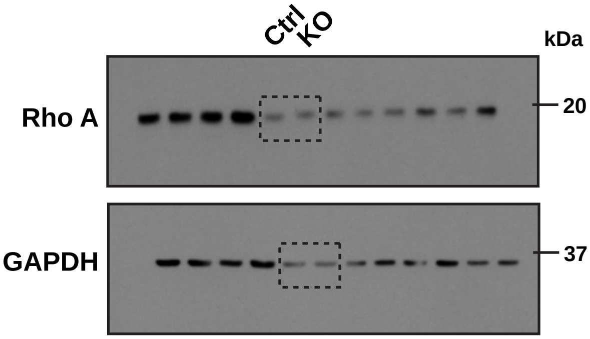

Supplementary Figure 6c

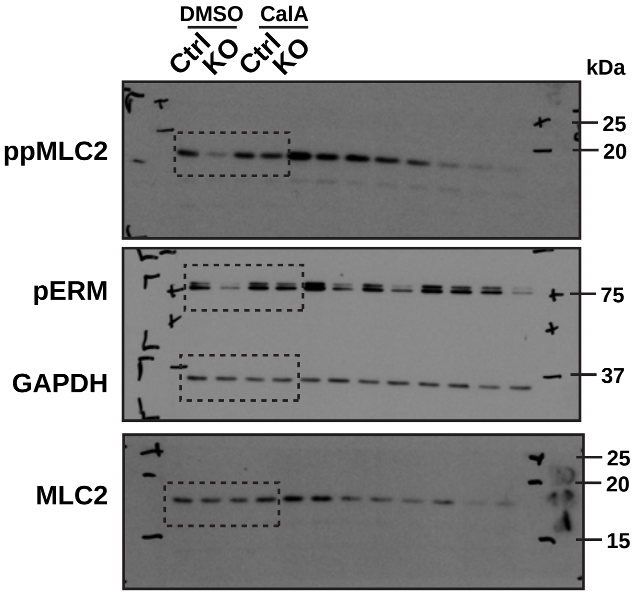

Supplementary Figure 8b

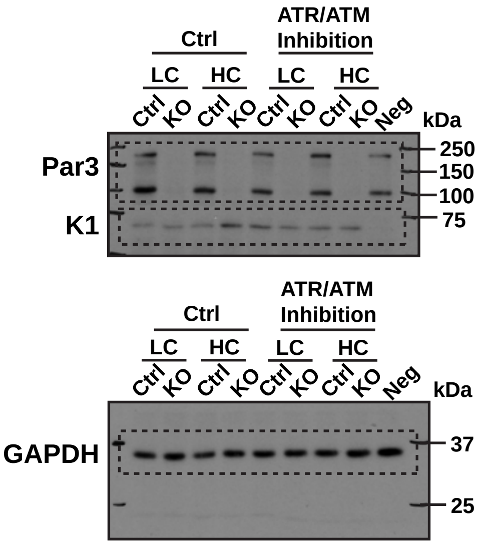

Supplementary Figure 12: Original western blots of the manuscript (3 of 3). Left vertical lines refer to the part of the membrane that was incubated with the antibody indicated next to it.

Supplementary Table 1. Antibodies and imaging reagents used in this study.

Primary antibodies (supplier)	Catalog nr.	Clone/Ref.	Lot nr.	Species	Dilution
Alpha-Tubulin (Sigma)	T6793	B-5-1-2	034M4837	mouse	IF 1:1000
Beta-catenin (BD Pharmingen)	610154	C19220	14	mouse	IF:500
CD34 Monoclonal Antibody, Biotin-conj. (eBioscience)	13-0341-85	RAM34	E032254	rat	IF 1:50
Chk1 (Cell Signaling Techn.)	2360	2G1D5/ 10/2015	3	mouse	WB 1:1000
CPD (Cosmo Bio)	NM-DND-001	TDM-2	TM-C-08	mouse	SB 1:10000
GAPDH (Millipore)	MAB374	6C5	2322571	mouse	WB 1:18.000
γH2Ax (S139) (Cell Signaling Techn.)	9718	20E3/ 07/2017	13	rabbit	IF 1:500
Involucrin (Covance)	PRB 140C-200	Polyclonal	N/A	rabbit	WB 1:1000
Keratin-1 (Covance)	PRB-165P	Polyclonal	N/A	rabbit	WB 1:1000
Keratin-15 (Thermo Scientific)	MA1-90929	Monoclonal	PL1944821	mouse	IF 1:1000
MLC2 (Cell Signaling Techn.)	3672	Polyclonal/ 09/2017	4	rabbit	WB 1:1000
Par3 (Millipore)	07-330	Polyclonal	2615671	rabbit	IF 1: 300
Pericentrin (BioLegend)	923701	Poly 19237	B200667	rabbit	IF 1:300
pEzrin (Thr567)/Radixin (Thr567)/Moesin (Thr558) (Cell Signaling)	3726	48G2/ 01/2018	5	rabbit	IF 1:300<br>IHC 1:200
pEzrin (Thr567)/Radixin (Thr567)/Moesin (Thr558) (Cell Signaling Techn.)	3141	T558/ 04/2018	15	rabbit	WB 1:1000
p53 (Leica)	NCL-L-p53-CM5p	Polyclonal	6044554	rabbit	IF 1:200<br>WB 1:1000
P190 RhoGAP (BD Transduction Laboratories)	610150	monoclonal	7201860	mouse	WB 1:2000
RhoA (Cell Signaling Techn.)	2117T	67B9	5	rabbit	WB 1:1000
RhoA (Cytoskeleton Inc.)	GL501	N/A	005	mouse	GLISA 1:250
pATR (Ser428) (Cell Signaling Techn.)	2853	Polyclonal 02/2014	5	rabbit	WB 1:1000
pChk1 (Ser345) (Cell Signaling Techn.)	2348	133D3/ 09/2015	15	rabbit	WB 1:1000
pMLC2 (Ser19) (Cell Signaling Techn.)	3675	Polyclonal/ 09/2016	5	mouse	IF 1:300<br>IHC 1:200
ppMLC2 (Thr18/Ser19) (Cell Signaling Techn.)	3674	Polyclonal/ 10/2017	5	rabbit	WB 1:1000<br>IHC 1:100
Survivin (Cell Signaling Techn.)	2808	71G4B7	N/A	rabbit	IF 1:100
**Secondary antibodies for immunofluorescence analyses**					
AlexaFluor 488 α-mouse (Invitrogen)	A21202	Polyclonal	1741782	donkey	IF 1:500
AlexaFluor 488 α-rabbit (Invitrogen)	A21206	Polyclonal	1910751	donkey	IF 1:500
AlexaFluor 568 α-rabbit (Invitrogen)	A11036	Polyclonal	1832035	goat	IF 1:500
AlexaFluor 568 α-rat (Invitrogen)	A11077	Polyclonal	870966	goat	IF 1:500
AlexaFluor 594 α-mouse (Invitrogen)	A21203	Polyclonal	1165596	donkey	IF 1:500
AlexaFluor 594 α-rabbit (Invitrogen)	A21207	Polyclonal	N/A	donkey	IF 1:500
AlexaFluor 647 α-mouse (Invitrogen)	A21235	Polyclonal	N/A	goat	IF 1:500
AlexaFluor 647 α-rabbit (Invitrogen)	A31573	Polyclonal	N/A	donkey	IF 1:500
**Secondary antibodies for immunoblot analyses**					
HRP α-rabbit (GE Healthcare)	NA9340V	Polyclonal	9720820	donkey	WB 1:4000
HRP α-mouse (GE Healthcare)	NA931V	Polyclonal	11076057	sheep	WB 1:4000
**Other Reagents**					
DAPI (Roth)	D1306	N/A	N/A	N/A	2μg/ml
HRP α-mouse (Cytoskeleton Inc.)	GL02	N/A	055	N/A	GLISA 1:62.5
**iFISH probes:** Tlk2 (11qE1) / Aurka (2qH3) Mouse probe (Leica)	KBI-30501	N/A	59325	DNA probe	1:1

Supplementary Table 2.
TaqMan® Gene Expression Assays (ThermoFisher Scientific) used in this study.

Target gene	ID	Cat. No.	label
Tgm2	Mm00436979_m1	4448892	Fam
Dvl1	Mm00438592_m1	4448892	Fam
Serpinf1	Mm00441270_m1	4448892	Fam
Rpa2	Mm00488047_m1	4448892	Fam
Ddit4l	Mm00513313_m1	4448892	Fam
Fas	Mm01204974_m1	4448892	Fam
Bmp7	Mm00432102_m1	4453320	Fam
Pmaip1 (Noxa)	Mm00451763_m1	4453320	Fam
Hprt	Mm00446968_m1	4448489	Vic
Mlh1	Mm00503449_m1	4448892	Fam
Msh2	Mm00500563_m1	4448892	Fam

Supplementary Table 3. Primers used in this study.

Name	Purpose	Sequence 5’-3’
CAAX hRas PCR 2 forw	Cloning/ CAAX PCR 2	CTT CTA CTC CCC TAG GGG CTG CAT GAG C
CAAX hRas PCR 2 rev	Cloning/ CAAX PCR 2	GCG GCC GCT CAG GAG AGC ACA CAC TTG CAG CTC ATG CAG CC
PCR1/3 fw NEU 160615	Cloning/ CAAX PCR 1+3	ACG GGA ACA TTC CTT TCC AC
PCR1 hRas CAAX rev NEU150615	Cloning/ CAAX PCR 1	AGC CCC TAG GGG AGT AGA AGG GCC G
PCR 3 hRas CAAX rev NEU150615	Cloning/ CAAX PCR 3	GCG GCC GCT CAG G
Cre-3	K14 Cre/ Genotyping	CGA TGC AAC GAG TGA TGA GGT TC
Cre-5	K14 Cre/Genotyping	GCA CGT TCA CCG GCA TCA AC
+598iR	Par3A deletion PCR/Genotyping	TAC CGT TAA CTG CAG CTC GGC TCT G
0509iR	Par3A deletion PCR/Genotyping	AGC TGG CGC TGG TAC CAT CTC CTC C
-423iF	Par3A Deletion, Flox PCR/Genotyping	AGG CTA GCC TGG GTG ATT TGA GAC C
-159iR	Par3A Flox PCR/Genotyping	TTC CCT GAG GCC TGA CAC TCC AGT C
