# Supplementary material for: Polarity signaling ensures epidermal homeostasis by coupling cellular mechanics and genomic integrity
Source: Nat Commun. 2019 Jul 29;10:3362. doi: 10.1038/s41467-019-11325-3 (PMC6662827; doi:10.1038/s41467-019-11325-3)
Supplement: Supplementary file 3 — Description of Additional Supplementary Files [file 41467_2019_11325_MOESM3_ESM.docx]

**Description of Additional Supplementary Files**

**File Name: Supplementary Movie 1**

**Description:** Live-cell imaging of primary control keratinocytes expressing H2B-GFP treated with DMSO. Videos were generated using a Leica® DMI 6000 microscope and a PlanApo 20x 0.75 NA oil objective. Scale bar: 50µm. Frame interval: 10 min; frame rate: 7fps.

**File Name: Supplementary Movie 2**

**Description:** Live-cell imaging of primary Par3KO keratinocytes expressing H2B-GFP treated with DMSO. Videos were generated using a Leica® DMI 6000 microscope and a PlanApo 20x 0.75 NA oil objective. Scale bar: 50µm. Frame interval: 10 min; frame rate: 7fps.

**File Name: Supplementary Movie 3**

**Description:** Live-cell imaging of primary control keratinocytes expressing H2B-GFP treated with Calyculin A. Videos were generated using a Leica® DMI 6000 microscope and a PlanApo 20x 0.75 NA oil objective. Scale bar: 50µm. Frame interval: 10 min; frame rate: 7fps.

**File Name: Supplementary Movie 4**

**Description:** Live-cell imaging of primary Par3KO keratinocytes expressing H2B-GFP treated with Calyculin A. Videos were generated using a Leica® DMI 6000 microscope and a PlanApo 20x 0.75 NA oil objective. Scale bar: 50µm. Frame interval: 10 min; frame rate: 7fps.

**File Name: Supplementary Movie 5**

**Description:** Live-cell imaging of primary control keratinocytes expressing H2B-GFP treated with CN03. Videos were generated using a Leica® DMI 6000 microscope and a PlanApo 20x 0.75 NA oil objective. Scale bar: 50µm. Frame interval: 10 min; frame rate: 7fps.

**File Name: Supplementary Movie 6**

**Description:** Live-cell imaging of primary Par3KO keratinocytes expressing H2B-GFP treated with CN03. Videos were generated using a Leica® DMI 6000 microscope and a PlanApo 20x.
