# Supplementary material for: Polarity signaling ensures epidermal homeostasis by coupling cellular mechanics and genomic integrity
Source: Nat Commun. 2019 Jul 29;10:3362. doi: 10.1038/s41467-019-11325-3 (PMC6662827; doi:10.1038/s41467-019-11325-3)
Supplement: Supplementary file 10 — Reporting Summary [file 41467_2019_11325_MOESM10_ESM.pdf]

nature research

Corresponding author(s): Sandra Iden

Last updated by author(s): Jun 12, 2019

Reporting Summary

Nature Research wishes to improve the reproducibility of the work that we publish. This form provides structure for consistency and transparency in reporting. For further information on Nature Research policies, see Authors & Referees and the Editorial Policy Checklist.

Statistics

For all statistical analyses, confirm that the following items are present in the figure legend, table legend, main text, or Methods section.

n/a	Confirmed	
☐	☒	The exact sample size (*n*) for each experimental group/condition, given as a discrete number and unit of measurement
☐	☒	A statement on whether measurements were taken from distinct samples or whether the same sample was measured repeatedly
☐	☒	The statistical test(s) used AND whether they are one- or two-sided<br>*Only common tests should be described solely by name; describe more complex techniques in the Methods section.*
☐	☒	A description of all covariates tested
☐	☒	A description of any assumptions or corrections, such as tests of normality and adjustment for multiple comparisons
☐	☒	A full description of the statistical parameters including central tendency (e.g. means) or other basic estimates (e.g. regression coefficient) AND variation (e.g. standard deviation) or associated estimates of uncertainty (e.g. confidence intervals)
☐	☒	For null hypothesis testing, the test statistic (e.g. *F*, *t*, *r*) with confidence intervals, effect sizes, degrees of freedom and *P* value noted<br>*Give P values as exact values whenever suitable.*
☒	☐	For Bayesian analysis, information on the choice of priors and Markov chain Monte Carlo settings
☒	☐	For hierarchical and complex designs, identification of the appropriate level for tests and full reporting of outcomes
☒	☐	Estimates of effect sizes (e.g. Cohen's *d*, Pearson's *r*), indicating how they were calculated

Our web collection on statistics for biologists contains articles on many of the points above.

Software and code

Policy information about availability of computer code

Data collection	Data collection utilized the following software: Microscopy: LASX (Leica), ZEN (Zeiss), Volocity (PerkinEllmer); Immunoblot: Samsung SCX3405 printer/scanner; GelDoc (Biorad); Atomic Force spectroscopy: JPK nanowizard; qRT-PCR: QuantStudio 12K Flex Software (Thermo)
Data analysis	For data analysis, the following software has been used: GraphPad PRISM VI, R, ImageJ/Fiji, Photoshop, MATLAB, PIV, CellProfiler, AtomicJ, GelAnalyzer

For manuscripts utilizing custom algorithms or software that are central to the research but not yet described in published literature, software must be made available to editors/reviewers. We strongly encourage code deposition in a community repository (e.g. GitHub). See the Nature Research guidelines for submitting code & software for further information.

Data

Policy information about availability of data

All manuscripts must include a data availability statement. This statement should provide the following information, where applicable:

- Accession codes, unique identifiers, or web links for publicly available datasets
- A list of figures that have associated raw data
- A description of any restrictions on data availability

The data that supports the findings of this study are provided in the main figures and supplementary figures, the published Source data file, or is available from the corresponding author upon reasonable request. The following figures have associated raw data:
Figure 1a-k, Figure 2a-g, Figure 3a-l, Figure 4a-f, Figure 5b-g, Figure 6a,c-i, Figure 7a-g, Suppl. Figure 1a-e, Suppl. Figure 2a,b; Suppl. Figure 3a; Suppl. Figure 4a,b; Suppl. Figure 5a-g; Suppl. Figure 6a-g; Suppl. Figure 7a-d; Suppl. Figure 8a,b; Suppl. Figure 9a,b; Suppl. Figure10-12.

Field-specific reporting

Please select the one below that is the best fit for your research. If you are not sure, read the appropriate sections before making your selection.

☒ Life sciences ☐ Behavioural & social sciences ☐ Ecological, evolutionary & environmental sciences

For a reference copy of the document with all sections, see nature.com/documents/nr-reporting-summary-flat.pdf

Life sciences study design

All studies must disclose on these points even when the disclosure is negative.

Sample size	Sample sizes are indicated for each experiment and mostly n=5 per assay. Independent of the complexity of the assay, for statistical evaluation a minimum of 3 experiments with independent biological replicates (n) per assay were initially performed and then statistically evaluated. The obtained data for assay-related variation were then used for implementation of further experiments, determination of technical replicates and required sample sizes.
Data exclusions	Experimental animals: Sick, underdeveloped or by any other means and pre-established criteria (e.g. score sheets) atypical animals were excluded from the analyses. In subsequent analyses, no samples were excluded.<br>Primary cell culture: Occasionally, primary keratinocyte cultures show poor attachment or slow growth rates. These isolations can be easily recognized and were excluded from further analyses.<br>Subsequent analyses: Technical assessment in terms of staining specificity, transfer efficiency for immunoblots etc. was each time performed, whereby positive (where available) and negative controls were used for evaluation.
Replication	(n= mentioned in each figure legend for each assay)<br>For tissue analyses, experimental animals of different litters and dissected at different days have been analysed to replicate the findings reported in this study. For experiments employing primary keratinocytes, each experimental set was repeated with at least 3 independent keratinocyte isolates. Each experiment was performed from independent isolates and on different days, unless otherwise stated. Similar results were obtained across all repeats. For antibody validation purposed, skin explant studies were replicated twice.
Randomization	Mice of different genotypes were randomly co-housed. Males and females were used for experiments, whereby its ratio was kept comparable between test and control groups. Mice of different litters derived from different parents were included in the analysis. For cell culture studies, different well positions and formats, and different incubators were measures of randomization. Moreover, primary cells were isolated from several litters that additionally had different parents (see also methods section).
Blinding	Investigators were not blinded to data collection and analyses. However, automated analyses was employed where possible.

Reporting for specific materials, systems and methods

We require information from authors about some types of materials, experimental systems and methods used in many studies. Here, indicate whether each material, system or method listed is relevant to your study. If you are not sure if a list item applies to your research, read the appropriate section before selecting a response.

Materials & experimental systems

n/a	Involved in the study	
☐	☒	Antibodies
☒	☐	Eukaryotic cell lines
☒	☐	Palaeontology
☐	☒	Animals and other organisms
☒	☐	Human research participants
☒	☐	Clinical data

Methods

n/a	Involved in the study	
☒	☐	ChIP-seq
☒	☐	Flow cytometry
☒	☐	MRI-based neuroimaging

Antibodies

Antibodies used	All information on antibodies used in this study is provided in Supplementary Table 1, including dilutions used.
Validation	In this study, only commercially available antibodies were used. Information on antibody validation is stated in the product data sheets provided by the supplier. For DNA damage-related proteins (pATR, pChk1, gammaH2Ax) UV-B or doxorubicin-treated keratinocytes served as positive control to validate specificity. For p53 antibodies used in Western Blotting, the correct band has been verified using lysates derived from either sip53-transfected and from p53KO cells. For BrdU antibodies, unlabeled samples were used as negative control to assess background binding. For all secondary antibodies used in immunofluorescence analyses, samples not incubated with primary antibodies were routinely taken along in experiments to examine potential background binding. Comprehensive validation experiments were performed for pMLC2 Ser19 (CST #3675; Suppl. Fig. 5 plus confidential data) to confirm its specificity. A step-by-step protocol for immunostaining of phosphorylated MLC2 in adult skin is provided at ProtocolExchange.

Animals and other organisms

Policy information about studies involving animals; ARRIVE guidelines recommended for reporting animal research

Laboratory animals	Live vertebrates (mice) were used in this study to obtain offspring of the required genotypes. From sacrificed animals, skin was dissected for further tissue analysis, or for isolation of primary murine keratinocytes. Mice were housed and fed according to federal guidelines. All breeding schemes and animal experiments were performed according to institutional guidelines and animal licenses of the State Office of North Rhine-Westphalia, Germany (Landesamt für Natur, Umwelt und Verbraucherschutz Nordrhein-Westfalen, LANUV).
Wild animals	The study did not involve wild animals.
Field-collected samples	The study did not involve field-collected samples.
Ethics oversight	No ethical approval was required for this study as not human research participants were involved in this study. Animal ethical review was integral part of the animal experiment licensing procedures (see above).

Note that full information on the approval of the study protocol must also be provided in the manuscript.
